# Genetic and Phenotypic Divergence in a Dung Beetle 50 Years After Its Introduction to Australia

**DOI:** 10.1002/ece3.70536

**Published:** 2024-11-10

**Authors:** Boikhutso Lerato Rapalai, Leigh W. Simmons, Theodore A. Evans, W. Jason Kennington

**Affiliations:** ^1^ Centre for Evolutionary Biology The University of Western Australia Crawley Western Australia Australia; ^2^ School of Biological Sciences The University of Western Australia Crawley Western Australia Australia

**Keywords:** *Digitonthophagus gazella*, introduced species, phenotypic adaptation, population structure, single nucleotide polymorphisms

## Abstract

Species translocations are increasingly being used in conservation and for biological control. The success of a translocation can be strongly influenced by the evolutionary processes occurring during the early phase of the introduction and the subsequent spread to new regions. In this study, morphological variation and population genetic structure were assessed in the African dung beetle *Digitonthophagus gazella,* a species that was intentionally introduced to Australia for biological control in 1968 and subsequently spread widely across the northern part of the continent. A dataset based on 1594 neutral single nucleotide polymorphism (SNP) loci that were genotyped in 187 individuals from 12 sites revealed significant genetic divergences between sites (global *F*_ST_ = 0.118) and provides evidence of restricted gene flow among established populations at small to moderate spatial scales (74–500 km). Geometric morphometric analyses revealed significant divergence among populations in the shape of the foretibia, a trait ecologically important for tunnelling in soil and dung. Moreover, phenotypic divergence in this trait for both sexes was significantly higher than genetic differentiation at selectively neutral loci (*P*_ST_ > *F*_ST_), suggesting that directional selection is contributing to the phenotypic divergences among populations. Our study shows how population structure can establish quickly in an introduced species and highlights the importance of considering local adaptation when performing translocations on established populations.

## Graphical abstract

Species translocations for conservation and biological control can be influenced by evolutionary processes occurring during the initial introduction and subsequent spread. This study assessed the morphological variation and population genetic structure of the African dung beetle, *Digitonthophagus gazella*, introduced to Australia in 1968, using data from 1594 SNP loci genotyped in 187 individuals from 12 sites. The findings revealed significant genetic divergence and restricted gene flow among populations, as well as significant phenotypic divergence in a key morphological trait due to directional selection, emphasising the importance of local adaptation in translocation efforts.

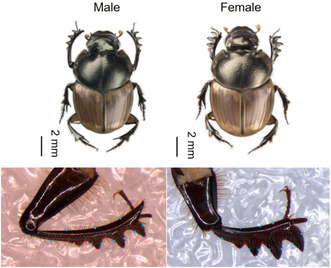

## 1 Introduction

Species translocations, the human‐aided movement of species from one location to another (IUCN 1987), are used effectively in conservation management (Griffith et al. 1989; Seddon, Strauss, and Innes 2012) as well as in classical biological control (Fauvergue et al. 2012; Roderick and Navajas 2003). When a species is translocated outside its historical range, it is subject to novel evolutionary processes because of the interaction between co‐adapted alleles and novel environmental conditions (Allendorf and Lundquist 2003; Pérez et al. 2006). Hence, evolutionary processes may either allow a translocated species to thrive or render it vulnerable to extirpation (Wright and Bennett 2018). As a result, an understanding of the species' population genetics and evolutionary ecology is necessary for monitoring and management, as well as for aiding intentional releases to increase chances of success in future translocation programmes.

As with other biological invasions, a translocated species must go through a crucial multistage process that encompasses three pivotal phases: introduction, establishment and expansion (Sakai et al. 2001). The introduction phase involves a series of deliberate human interventions, beginning with the selection or capture of individuals, a process analogous to artificial selection, followed by their transportation and subsequent release into a novel environment (Baños‐Villalba et al. 2021; Blackburn et al. 2011; Puth and Post 2005). Upon introduction, the species enters the establishment phase, wherein it must overcome various ecological barriers to form a stable, self‐sustaining population within the new habitat (Blackburn et al. 2011; Marsico et al. 2010; Sakai et al. 2001). If successful, the translocated species progresses to the expansion phase, where it disperses and spreads well beyond its introduction site (Allendorf and Lundquist 2003; Marsico et al. 2010; Sakai et al. 2001). Translocated populations are often established by a small number of individuals relative to native range populations (Allendorf and Luikart 2012; Griffith et al. 1989). This can result in a founder event, which leads to loss of allelic diversity and large fluctuations in allele frequencies (Nei, Maruyama, and Chakraborty 1975). Founder effects are exacerbated when translocated populations are geographically isolated from the source populations or when there is a barrier to dispersal between them (Tigano and Friesen 2016). Loss of allelic diversity is a concern as it has deleterious effects on newly established populations (Allendorf and Luikart 2012). Inbreeding depression is bound to occur, and the species undergoes reduction in fitness and a loss of evolutionary potential (Frankham 2005; Franks, Pratt, and Tsutsui 2011; Furlan et al. 2012), resulting in failure of a translocation.

A fundamental phase of a translocation is when a species' phenotype adapts in response to new selection pressures (Suarez and Tsutsui 2008). Adaptation can arise via two processes: genetic adaptation and phenotypic plasticity. Genetic adaptation occurs when a species responds to novel selection pressures whereby advantageous alleles increase in frequency to give rise to adaptive phenotypic traits (Orr 2005). Phenotypic plasticity refers to the ability of a single genotype to give rise to different phenotypes as a response to the demands of different environmental conditions (Allendorf and Luikart 2012; Price, Qvarnström, and Irwin 2003; West‐Eberhard 1989). Phenotypic plasticity in life history traits, including morphology, physiology and behaviour (Sommer 2020), can be manifest at the individual level (Ghalambor et al. 2007), impact different life stages (Stillwell and Fox 2005) and affect multiple generations (Donelson et al. 2018). Phenotypic plasticity can serve as a ‘rapid‐response mechanism’ facilitating long‐term genetic adaptation (Fox et al. 2019).

The interplay between genetic adaptation and phenotypic plasticity is crucial in determining the success of translocated species in novel environments (Zenni et al. 2014). Often, phenotypic plasticity provides an immediate buffer against environmental variability and strong selection during the introduction phase of a translocation, while genetic adaptation, occurring over longer timescales and subsequent generations, ensures persistence during establishment and expansion (Ghalambor et al. 2007; Jardeleza et al. 2022; Reznick and Ghalambor 2001). For example, White Sands pupfish (*Cyprinodon tularosa*) in New Mexico were found to exhibit both plasticity and genetic adaptation in body shape following translocations (Collyer et al. 2007). Pupfish from saline waterbodies were found to be more lean than pupfish from brackish waterbodies, and fish from populations translocated 30 years prior to the study displayed significant body shape divergence compared with populations translocated just 1 year prior to the study (Collyer et al. 2007). These findings suggest that plastic changes were responsible for facilitating long‐term evolutionary changes in body shape, and that plasticity and evolutionary divergence can facilitate rapid range expansion and ultimately, successful translocations.

Dung beetles in the tribe Onthophagini, especially the genus *Onthophagus*, have become model organisms for studying phenotypic plasticity and evolution (Moczek 2011), particularly within the context of rapid range expansion and adaptation to novel environments (Rohner, Jones, and Moczek 2024). For example, in the bull‐headed dung beetle *Onthophagus taurus* (Schreber, 1759), adaptive plasticity and divergences in morphological, ecological and physiological life history traits have all been documented (Leeson et al. 2020; Macagno, Beckers, and Moczek 2015; Moczek 1998; Moczek et al. 2002; Pizzo et al. 2008; Rohner and Moczek 2020, 2023). This species was translocated to Australia as a biocontrol agent and introduced accidentally to the eastern United States, and has established successfully in both geographic regions (Hoebeke and Beucke 1997; Rounds and Floate 2012; Tyndale‐Biscoe 1996). Both exotic populations are similar in body size; however, they have diverged in the critical body size threshold at which males develop horns (Moczek et al. 2002). Moreover, these exotic populations appear to have diverged in both the depth at which they bury their broods, and in their foretibia morphology (Macagno, Moczek, and Pizzo 2016).

The introduction of dung beetles into Australia approximately 50 years ago marked one of the greatest successes in species translocations for biological control purposes (Bornemissza 1976; Edwards 2007; Leeson et al. 2020). Before these introductions, livestock dung fouled pastures by making them unpalatable and provided a breeding site for fly pests and associated parasites (Bornemissza 1970; Davis 1987; Doube 2018). The Commonwealth Scientific and Industrial Research Organisation (CSIRO) launched the Dung Beetle Project to kick start dung beetle translocations in 1964 (Bornemissza 1976). One of the criteria the CSIRO used in selecting species suitable for translocations was to identify those with a wide geographic distribution and thus the potential for different populations of a species to have a broad spectrum of climatic and ecological adaptations (Bornemissza 1976), what the CSIRO referred to as ‘strains’ (Edwards 2007). Capturing and releasing different strains was fundamental in ensuring sufficient genetic variation, thereby providing the flexibility needed for adaptation to various environmental conditions in novel Australian habitats (Bornemissza 1976). One of the onthophagine dung beetle species introduced to Australia that became established was the African dung beetle, *Digitonthophagus gazella* (Fabricius, 1787). The ‘tropical’ strain of *D. gazella* was the first to be released in Australia in 1968, and was sourced from the tropical region east of sub‐Saharan Africa (Zimbabwe, Kenya, Mozambique and South Africa) via Hawaii (Bornemissza 1976; Edwards 2007; Markin and Yoshioka 1998), with the release conducted in Townsville in northern Queensland (Edwards 2007). Three more strains from different parts of sub‐Saharan Africa were subsequently released between 1970 and 1984 (Bornemissza 1976; Edwards 2007). The ‘gene pool’ strain, anticipated to be more ‘genetically enriched’, was sourced from a variety of different populations from southern Africa (Mozambique, Malawi, South Africa and Zimbabwe) (Bornemissza 1976), and released in northern tropical Australia (Edwards 2007). The ‘even rainfall’ strain was sourced from subtropical regions south east of the Eastern Cape in South Africa (Génier and Davis 2017) and was released in eastern New South Wales (Edwards 2007), and the ‘cold’ strain sourced from South Africa (Edwards 2007) was released in southern Australia (Bornemissza 1976; Edwards 2007). A total of 420,415 *D. gazella* beetles were released during that period (Edwards 2007). Due to high dispersal rates (Seymour 1980; Whipple et al. 2012) and multiple generations per year (Bornemissza 1976), this species expanded its range across an environmentally heterogenous landscape within a 50‐year time frame (Noriega et al. 2020).

Recent taxonomic research shows that the genus *Digitonthophagus* is made up of 16 cryptic species (Génier and Moretto 2017), instead of two morphologically variable species (*D. gazella* and *D. bonasus*) as was previously thought. This discovery raises the possibility that more than one *Digitonthophagus* species may have been introduced into Australia; one of the ‘strains’ subsequently introduced could have been a different species of *Digitonthophagus*. This possibility could explain why *D. gazella* thrived in the arid regions south of the Northern Territory, where BIOCLIM models had predicted an unfavourable area for establishment (Edwards 2007; Floate et al. 2017). Indeed. *D. gazella* was thought unable to establish in arid conditions with rainfall below 250 mm (Bornemissza 1970; Génier and Moretto 2017). Although species characterisation based on assessments of male genitalia contends that the species introduced widely in Australia is *D. gazella* (Noriega et al. 2020), it is also possible that the species thriving in a supposedly unfavourable region is another species in the genus, perhaps *D. namaquensis* as this species' distribution extends into the Namib Desert in southern Africa (Génier and Moretto 2017). Nonetheless, it is also possible that *D. gazella* has become locally adapted to the arid regions of Australia, as the species is known to be resilient and tolerant to a broad range of climatic conditions (de Oca and Halffter 1995; Pokhrel, Cairns, and Andrew 2020). Here we use genetic and morphometric analyses to explore these alternative scenarios.

*Digitonthophagus gazella* belongs to a group of dung beetles known as tunnellers (paracoprids), characterised by digging tunnels and storing brood balls in the soil beneath the dung pad (Simmons and Ridsdill‐Smith 2011). As an adaptation to their burrowing (fossorial) nature, tunnellers have developed short, thick, flattened and toothed foretibiae mainly adapted for tunnelling through soil and dung (Linz, Hu, and Moczek 2019; Macagno, Moczek, and Pizzo 2016; Scholtz, Davis, and Kryger 2009; Simmons and Fitzpatrick 2019). This digging tool has enabled dung beetles to inhabit and adapt to an ecological niche that cannot be accessed by other insect taxa (Linz, Hu, and Moczek 2019). Both the male and female are active in dung provisioning for brooding; females spending more time digging tunnels and packing brood balls (Bornemissza 1970; Simmons and Fitzpatrick 2019), while males use their foretibia to assist females with packing brood balls (Bornemissza 1970; Simmons and Ridsdill‐Smith 2011). In onthophagines, the divergence in foretibia morphology has been attributed to climatic, environmental and ecological factors such as temperature‐dependent maternal care, type and moisture content of soil, as well as brood burial behaviour (Macagno, Moczek, and Pizzo 2016; Macagno et al. 2018; Rohner and Moczek 2020). We used geometric morphometric analysis to quantify divergence in foretibia morphology across the geographic range of *D. gazella* in Australia and contrast phenotypic divergence with genetic divergence.

To date, few population genetic studies have been conducted on *D. gazella* and none on the introduced Australian population. A study by Martins and Contel (2001) using esterase isozymes reported low levels of genetic variation in an introduced population established in Uberaba, Brazil. In another study using Amplified Fragment Length Polymorphisms (AFLPs), genetic variation was compared between introduced populations on the island of Vieques (Puerto Rico) and their source population from South Africa, and reported surprisingly high levels of genetic diversity in both the introduced and source populations, but little gene flow between them (Whipple et al. 2012). Here, we build on this work by investigating genetic variation in *D. gazella* across the Australian continent using single nucleotide polymorphisms (SNPs).

Our study had three aims: (1) to evaluate levels of phenotypic and genetic variation within and among Australian populations of *D. gazella*, (2) assess whether phenotypic divergences among Australian populations have arisen through natural selection or neutral genetic processes. Given the possibility that multiple cryptic *Digitonthophagus* species could have been introduced to Australia, we also sequenced the mitochondrial Cytochrome c oxidase subunit I (COI) gene to (3) verify the taxonomy of beetles collected from each of the sites sampled for our study.

## 2 Materials and Methods

### 2.1 Sample Selection

**FIGURE 1:**
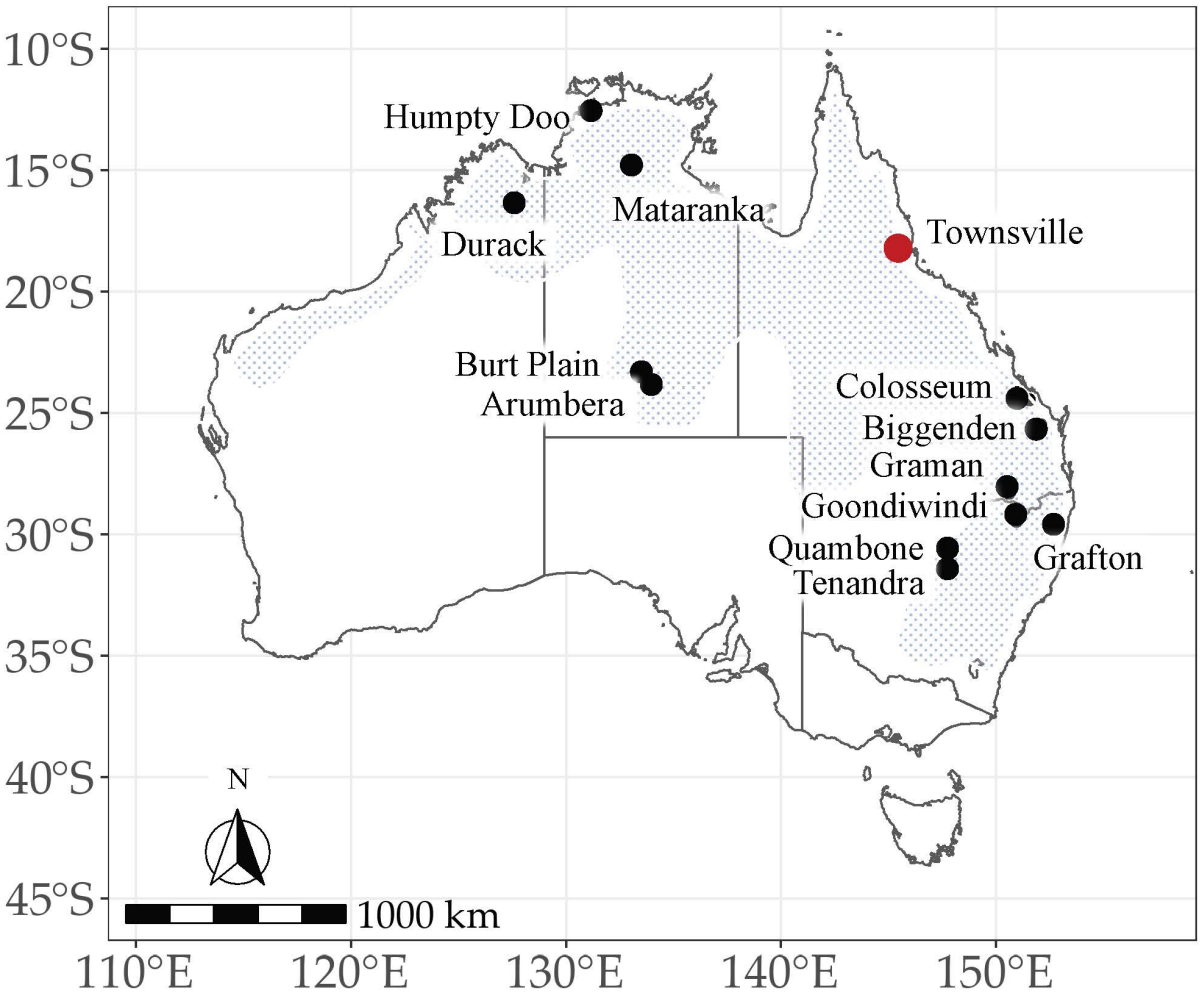
Sampling sites and distribution of *Digitonthophagus gazella* in Australia. The initial release site (Townsville) is indicated by the red symbol.

*Digitonthophagus gazella* were collected from 12 geographically distinct sites in Australia. Three sites were from the northern tropical region of the Northern Territory (NT) and Western Australia (WA), and two sites were from the southern arid region of the NT. The remaining sites were from the eastern subtropical region of Australia: three sites from south‐eastern Queensland (QLD), two sites from northern New South Wales (NSW) and two sites from the central regions of NSW (Figure 1). Samples were collected in January and February 2019 by local landowners and sent to the University of Western Australia for processing, where they were preserved in 100% ethanol. The number of specimens per site ranged from 12 to 30 (Table 1). No permits were required for collecting the beetles.

**TABLE 1:** Estimates of genetic diversity within populations.

Population	Location	Climatic region	*N*	*H*_e_	*H*_o_	*F*_IS_
Arumbera (AR)	Southern NT	Arid	30	0.212 (0.005)	0.185 (0.004)	0.107 (0.007)
Biggenden (BG)	Southern Qld	Subtropical	15	0.243 (0.004)	0.221 (0.005)	0.088 (0.008)
Burt Plain (BP)	Southern NT	Arid	15	0.209 (0.005)	0.185 (0.005)	0.100 (0.009)
Colosseum (CL)	Southern Qld	Subtropical	14	0.227 (0.005)	0.195 (0.005)	0.125 (0.010)
Durack (DR)	Northern WA	Tropical	14	0.225 (0.005)	0.193 (0.005)	0.126 (0.010)
Goondiwindi (GD)	Southern Qld	Subtropical	15	0.225 (0.004)	0.205 (0.004)	0.073 (0.008)
Grafton (GF)	Northern NSW	Subtropical	15	0.199 (0.005)	0.167 (0.004)	0.135 (0.009)
Graman (GM)	Northern NSW	Subtropical	14	0.208 (0.005)	0.184 (0.005)	0.105 (0.009)
Humpty Doo (HD)	Northern NT	Tropical	14	0.222 (0.005)	0.191 (0.005)	0.128 (0.010)
Mataranka(MT)	Northern NT	Tropical	15	0.221 (0.005)	0.190 (0.005)	0.128 (0.010)
Quambone (QB)	Central NSW	Subtropical	12	0.200 (0.005)	0.165 (0.005)	0.144 (0.011)
Tenandra (TN)	Central NSW	Subtropical	15	0.198 (0.005)	0.172 (0.005)	0.110 (0.010)

*Note:* Standard errors are in parentheses.

Abbreviations: *F*_IS_ = inbreeding coefficient, *H*_e_ = expected heterozygosity, *H*_o_ = observed heterozygosity, *N* = sample size.

### 2.2 Geometric Morphometric Analysis

**FIGURE 2:**
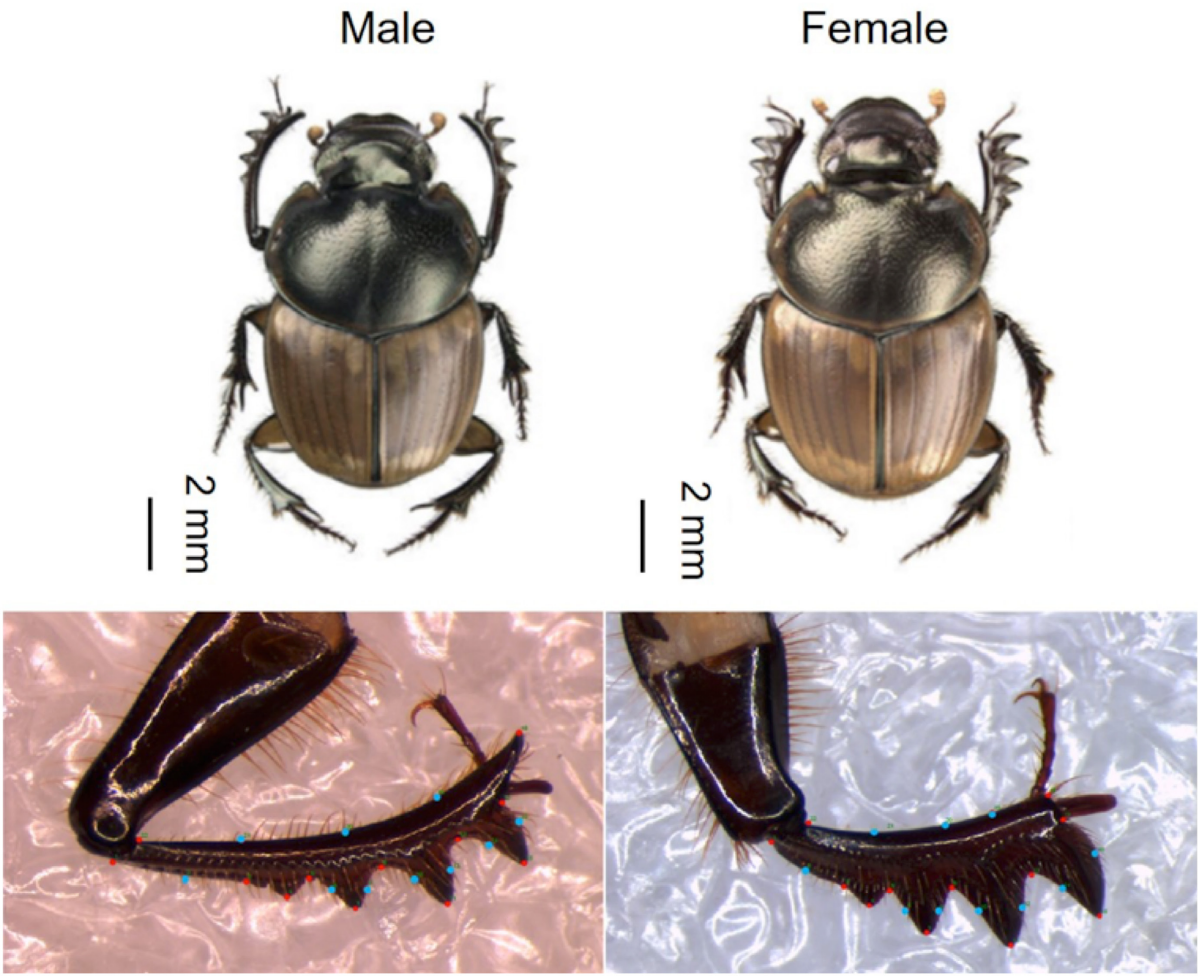
Sexual dimorphism displayed in *D. gazella* foretibia. Note the slender and elongated foretibia of the male, and the thicker foretibia of the female. Whole beetle images provided by Fançois Génier CC BY 3.0, from Génier and Davis (2017). The red dots on the images of foretibia indicate fixed landmarks and the blue dots indicate semi‐sliding landmarks used in the morphometric analysis.

Geometric morphometric analysis was employed to test for morphological divergence in the shape of the foretibia. Geometric morphometric analysis is a ‘landmark digitization’ based approach that involves obtaining shape variables to compute a consensus shape by superimposing all images of a structure (Zelditch, Swiderski, and Sheets 2004). In common with other onthophagines (Simmons and Fitzpatrick 2019), *D. gazella* exhibits sexual dimorphism in the shape of the foretibia. Males have a longer and thinner foretibia, while females have a shorter and more robust foretibia (Figure 2). As such, foretibia were separated by sex for analysis.

The male foretibia (*n*_total_ = 135) and female foretibia (*n*_total_ = 108) were dissected and mounted on microscope slides. Images of the structures were captured using a Leica DFC 290 camera fitted onto a Leica MZ7.5 high‐performance stereomicroscope. Care was taken to capture images in the same orientation. For both sexes, 22 landmarks (12 fixed and 10 semi‐sliding) were placed around the tibial teeth of the foretibia (Figure 2) using the software tpsDig2v2 (Rohlf 2017). Imaging and landmarking were conducted by the same individual (BLR) to minimise measurement error.

Relative warps (RW) and centroid sizes (CS) were extracted in tpsRelw version 3.2 (Rohlf 2017). Relative warps were computed to describe the variation of each structure's shape relative to the consensus shape (Adams, Otárola‐Castillo, and Paradis 2013). CS is a proxy for the size of the structure, obtained by calculating the square root of the sum of squared distances between each landmark position and the average position of the centroid (Zelditch, Swiderski, and Sheets 2004). To ensure consistency and test repeatability, landmarks were placed on three replicate images of the same individual, for the foretibia of 10 males and 10 females. The centroid size and the first three relative warps were then subjected to a repeatability analysis using the IRR package in R, by estimating the inter‐class correlations using the two way model (Gamer 2010). Measurements were highly repeatable for both female (RW 1: *R* = 0.868, *p* < 0.01; RW 2: *R* = 0.447, *p* < 0.01; RW 3: *R* = 0.723, *p* < 0.001; centroid size: *R* = 0.994, *p* < 0.001) and male foretibia (RW 1: *R* = 0.986, *P* ≤ 0.001; RW 2: *R* = 0.882, *p* < 0.001; RW 3: *R* = 0.808, *p* < 0.001, centroid size: *R* = 0.986, *p* < 0.001). A visual representation of shape variations for all structures was constructed using Thin Plate Splines (TPS) in the tpsRelw version 3.2 (Rohlf 2017).

Shape variation among populations and among climatic regions was tested using Multivariate Analysis of Covariance (MANCOVA) controlling for CS, because CS was correlated with RW1 (Pearson's *r* = 0.89, *p* < 0.001) and RW2 (Pearson's *r* = −0.20, *p* = 0.018) in males, and RW3 (Pearson's *r* = −0.64, *p* < 0.001) in females. Pairwise differences between populations and between climatic regions (tropical, subtropical and arid) were assessed using univariate post hoc Tukey‐HSD tests.

### 2.3 DNA Extraction and COI Sequencing

The cytochrome c oxidase subunit I (COI) mitochondrial gene region was utilised to further explore the species status of introduced populations of *Digitonthophagus*. This gene region was chosen because it displays rapid rates of evolution and is also present in numerous copies (Schwarzfeld and Sperling 2014). In dung beetle taxonomy and systematics, COI sequencing has been utilised successfully to delimit species and infer phylogenies such as across the African Scarabaeinae family (Mlambo, Sole, and Scholtz 2015), the tribe Onthophagini (Roggero, Barbero, and Palestrini 2017; Tarasov and Solodovnikov 2011) and the genus *Onthophagus* (Emlen et al. 2005).

DNA was extracted from three right side legs of each dung beetle specimen using the DNeasy Blood and Tissue Kit (Qiagen Inc., USA) following the manufacturer's instructions. In total, 60 DNA extractions were completed for five individuals per population. A 612 bp fragment of the mitochondrial COI gene region was amplified using Polymerase Chain Reaction (PCR) with the primers LCO1490 and HCO2198 (Folmer et al. 1994). Samples were sent to the Australian Genome Research Facility (AGRF) for bi‐directional sequencing of PCR products performed via the Sanger sequencing approach. Sequences were trimmed and edited using the Geneious Prime software (http://www.geneious.com) (Kearse et al. 2012). For each individual, species identification was verified by similarity‐based searches on the Barcode of Life Database (BOLD) and the NCBI BLAST database (Altschul et al. 1990). Sequences were also compared to four *D. gazella* (accession numbers: MN345971.1, EU162450.1, HQ559831.1, HQ559816.1), one *Onthophagus taurus* (Accession number: EU162476.1), one *Onthophagus diabolicus* (accession number: KU739483.1), one *Phalops ardea* (accession number: KU739473.1) and one *D. bonasus* (accession number: KU739459.1) obtained from the NIH genetic sequence database (GenBank, https://www.ncbi.nlm.nih.gov/genbank/). There was no genomic information for other *Digitonthophagus* species except for *D. bonasus*, which is widely distributed in Asia (Génier and Moretto 2017).

### 2.4 Genetic Assessment of Number of Species

To infer a phylogeny for the sampled populations, a Maximum Likelihood Tree was constructed using IQ‐TREE (Nguyen et al. 2015), via the iq‐tree Web server (http://iqtree.cibiv.univie.ac.at/). The Maximum Likelihood Tree was constructed using 1000 ultrafast bootstrap alignments (Hoang et al. 2018), 1000 iterations and 0.99 minimum correlation coefficient. The IQ‐TREE selected ‘GTR + F + I + G4’ as the best fit model of partition (Kalyaanamoorthy et al. 2017). The phylogenetic tree was visualised via treeview v3.2 software (Page 1996). To further evaluate the identity of species, intraspecific and interspecific COI sequence divergence values were calculated using the pairwise distance matrix in geneious prime (http://www.geneious.com) (Kearse et al. 2012). A haplotype network was constructed using the *haploNet* function in the R package PEGAS (Paradis 2010).

### 2.5 SNP Genotyping

Tissue samples from 188 individuals (*n*_females_ = 86, *n*_males_ = 102) were sent to Diversity Arrays Technology (https://www.diversityarrays.com/) for DNA extraction and SNP genotyping using the DArTseq approach. DArTseq merges complexity reduction processes with next generation sequencing to produce a genome wide data set of SNP markers (Kilian et al. 2012). Four complexity reduction enzyme combinations were tested. Based on these tests, the *Pst*I*‐Sph*I enzyme combination was selected and used for the DNA digests. Small fragments of digested DNA were subsequently ligated to barcoded adaptors. The forward cutter adaptor included an Illumina flow cell attachment sequence, sequencing primer sequence and barcode region. The reverse adaptor has two regions: the illumina flow cell attachment and a compatible overhang sequence. Digestion/ligation reactions were used to process the DNA fragments (Kilian et al. 2012). Samples were then amplified through PCR, and the PCR products were standardised in concentration and pooled for sequencing. The pooled amplified samples were sequenced in Illumina HiSeq2500 and processed using DArTseq Analytical Pipelines.

Single nucleotide polymorphism data were filtered using the DARTR R package (Gruber et al. 2018), based on the following criteria: loci with a call rate less than 95%, individuals with a call rate less than 85%, loci with a minor allele frequency (MAF) of less than 0.05, monomorphic loci and one SNP locus per sequence. To generate a set of putatively neutral SNP loci, we screened our data for outlier loci (loci potentially under directional selection) using three different methods: OUTFLANK, BAYESCAN and PCADAPT.

OUTFLANK detects loci undergoing spatial heterogenous selection using an *F*_ST_ outlier approach (Whitlock and Lotterhos 2015). BAYESCAN uses a Bayesian model approach to calculate the posterior probability that each locus is subject to selection, assuming a Dirichlet distribution across allele frequencies (Foll and Gaggiotti 2008). PCADAPT uses a principal components approach to detect outlier loci (Luu, Bazin, and Blum 2017). The three methods were used for robustness and for reducing the likelihood of false positives. For instance, PCADAPT is known to have low false discovery rate than BAYESCAN (Luu, Bazin, and Blum 2017). Loci identified as outliers in any two of these methods were considered to be under directional selection and were removed from the data consisting of putatively neutral loci.

### 2.6 Population Structure and Genetic Diversity

Population structure was assessed using Discriminant Analysis of Principal Components (DAPC) (Jombart, Devillard, and Balloux 2010), Bayesian clustering analysis using STRUCTURE v2.3.4 (Hubisz et al. 2009) and by calculating pairwise *F*_ST_ values (Weir and Cockerham 1984). DAPC was conducted using the R package ADEGENET (Jombart 2008). Unlike the Principal Components Analysis (PCA), the DAPC was used as it is known to maximise variation between groups, while minimising variation within groups (Miller, Cullingham, and Peery 2020). A priori information was not used for assigning genetically related individuals into clusters. The cross‐validation method was used for retaining a mean of 40 principal components for the DAPC. The optimum number of clusters (*k*) was selected based on the Bayesian Information Criterion (Jombart, Devillard, and Balloux 2010). The analysis using STRUCTURE was based on an admixture model assuming correlated allele frequencies with 10,000 burn‐in cycles,100,000 MCMC replicates, and 10 repetitions for each value of K ranging from 1 to 10. The results from this analysis were represented visually using Clumpak (Kopelman et al. 2015) and the optimal number of genetic clusters determined using the delta K approach (Evanno, Regnaut, and Goudet 2005) with STRUCTURE HARVESTER (Earl and vonHoldt 2012). Global and pairwise *F*_ST_ were calculated using the R package HIERFSTAT (Goudet 2005). The significance of *F*_ST_ values was determined by calculating 95% confidence intervals with bootstrapping (1000). The relationship between geographical distance (log transformed) and genetic divergence was assessed using a Mantel test implemented using the R package ECODIST (Goslee and Urban 2007).

Estimates of observed and expected heterozygosity within populations and the inbreeding coefficient (*F*_IS_) were calculated using the R package HEIRFSTAT (Goudet 2005). A Kruskal‐Wallis test was used to detect whether expected heterozygosity differed significantly between populations. Dunn's non‐parametric all pair comparison test (Dunn 1964) was used to detect which population pairs differed significantly, correcting for multiple comparisons with the Benjamin‐Hochberg method (Benjamini and Hochberg 1995). The relationship between genetic diversity and distance to the first release site (Townsville) was assessed using regression analysis.

### 2.7 Patterns of Selection Underlying Phenotypic Divergence

In evolutionary studies, the degree of neutral genetic divergence, Wright's *F*_ST_ index (Wright 1931) is compared with the degree of phenotypic divergence, *Q*_ST_ index (Spitze 1993) to reveal patterns of selection driving phenotypic variation among populations. If *Q*_ST_ > *F*_ST_, directional selection is thought to be driving divergence across populations by favouring different phenotypic traits, leading to local adaptation (Defaveri and Merilä 2013). If *Q*_ST_ < *F*_ST_, stabilising selection is thought to be responsible for homogenising a phenotypic trait value across populations (Da Silva and Da Silva 2018). If *Q*_ST_ = *F*_ST_, divergence is assumed to have occurred due to genetic drift (Marin et al. 2018). Practically, the application of quantitative trait divergence (*Q*_ST_) is only possible in common garden experiments where environmental conditions are controlled. Therefore, the phenotypic trait index (*P*_ST_) (Brommer 2011; Leinonen et al. 2006) is used as a proxy for *Q*_ST_ for analysis of wild populations. The phenotypic trait index is calculated using the formula:

$$P_{\mathrm{ST}}=\frac{c}{h^{2}}{\sigma^{2}}_{B}/\left(\frac{c}{h^{2}}\;{\sigma^{2}}_{B+}\;2{\sigma^{2}}_{W}\right).$$

where *c* is the total variance caused by additive genetic effects, and *h* ^*2*^ is a measure of heritability (Brommer 2011). RWs were used as quantitative trait measures. To account for variations in trait shape that is correlated with trait size, RWs were corrected for CS using the simple linear adjustments transformation in R (Da Silva and Da Silva 2018). The transformed quantitative trait measures were used to calculate *P*_ST_ across populations using the R package PSTAT (Da Silva and Da Silva 2018). We used the function ‘*TracePst*’, which plots the *P*_ST_ of a given trait against a range of values of *c/h* ^*2*^, to test whether the global *P*_ST_ for each trait was higher than the global *F*_ST_. Pairwise *P*_*ST*_ values were calculated assuming a robust *c/h* ^*2*^ value of 1 (this assumes that the proportion of the total variance due to additive genetic effects across populations is equal to the heritability, *c = h* ^*2*^) and were compared to pairwise *F*_ST_ values using Mantel tests with the R package ecodist (Goslee and Urban 2007). Note that body size is strongly affected by environmental variation (Schwab and Moczek 2016), so that controlling for trait size serves to reduce variance in *P*_ST_ that might be due to environmental effects.

## 3 Results

### 3.1 Species Status Based on COI Sequences

**FIGURE 3:**
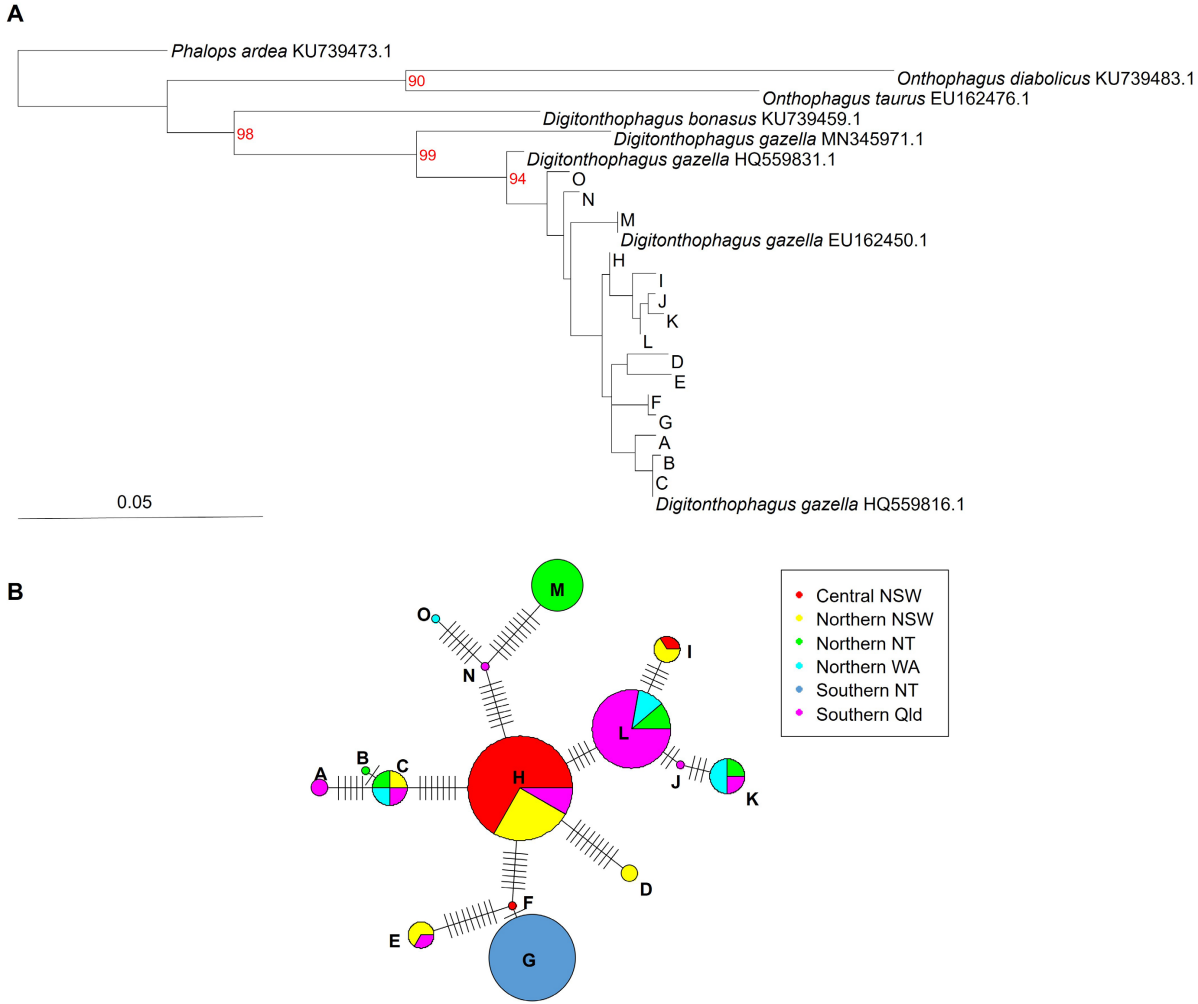
Evolutionary relationships among mitochondrial COI sequences. (A) Maximum Likelihood Tree with posterior probabilities above branches. Letters (A–M) denote the distinct genetic clade within sampled *D. gazella* populations, with each letter grouping individuals that share significant genetic similarity. (B) Haplotype network depicted by coloured pie charts. Each pie chart shows the geographic origin of haplotypes, with colours corresponding to different regions. The size of each circle represents the frequency of the haplotype, and branches indicate the number of mutational steps between haplotypes. Letters refer to the clade clusters in part A.

Fifteen unique haplotypes were observed among the 60 sequences generated in our study (accession numbers: OP364847–OP364861) with 42 polymorphic sites. The phylogenetic relationships among all sampled individuals and the sequences derived from GenBank are depicted in Figure 3. All of the sequences obtained in this study and three of the *D. gazella* GenBank sequences formed a single well‐supported monophyletic clade (Figure 3). The fourth *D. gazella* sequence retrieved from GenBank (accession number MN345971.1) fell outside this clade, but was the next most closely grouped sequence. All of the sequences obtained from other species formed their own separate clade (Figure 3).

Pairwise divergences between sequences from the sampled populations ranged from 0% to 2.8% with a mean value of 1.3% (Table A1 in the Appendix 1). These values were comparable with or lower than the pairwise divergences between *D. gazella* sequences retrieved from GenBank (range 2.1%–7.5%) and much lower than the pairwise divergences between species within the genus (range 11.2%–11.8%) and the tribe Onthophagini (11.5–17.3, Table A1 in the Appendix 1).

### 3.2 Morphological Divergence in Foretibia

**FIGURE 4:**
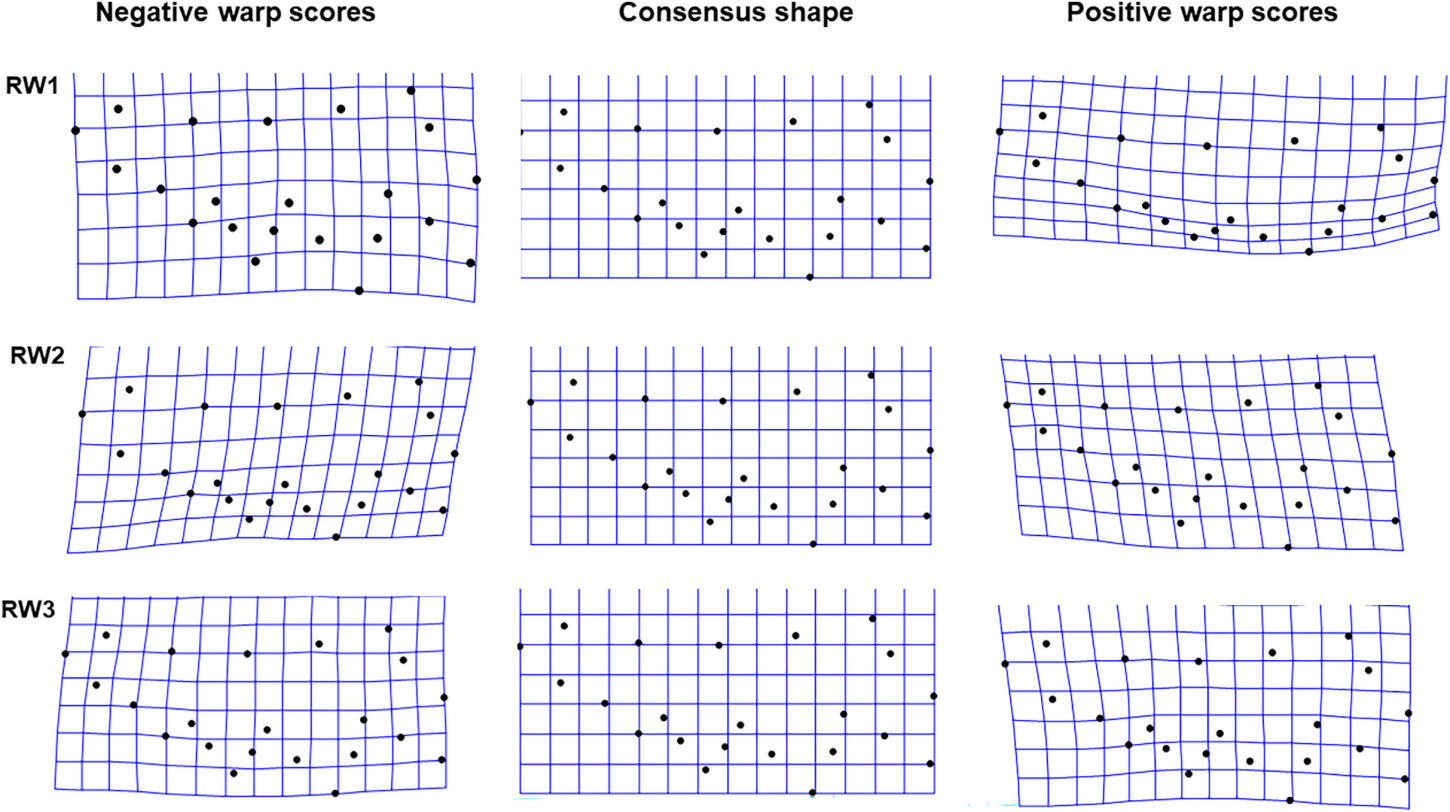
Thin plate splines showing shape variation as explained by RW1, RW2 and RW3 of the female foretibia. Black dots show position of landmarks. Top panel indicates RW1 shape variation, middle panel indicates RW2 shape variation, while bottom panel indicates RW3 shape variation.

**FIGURE 5:**
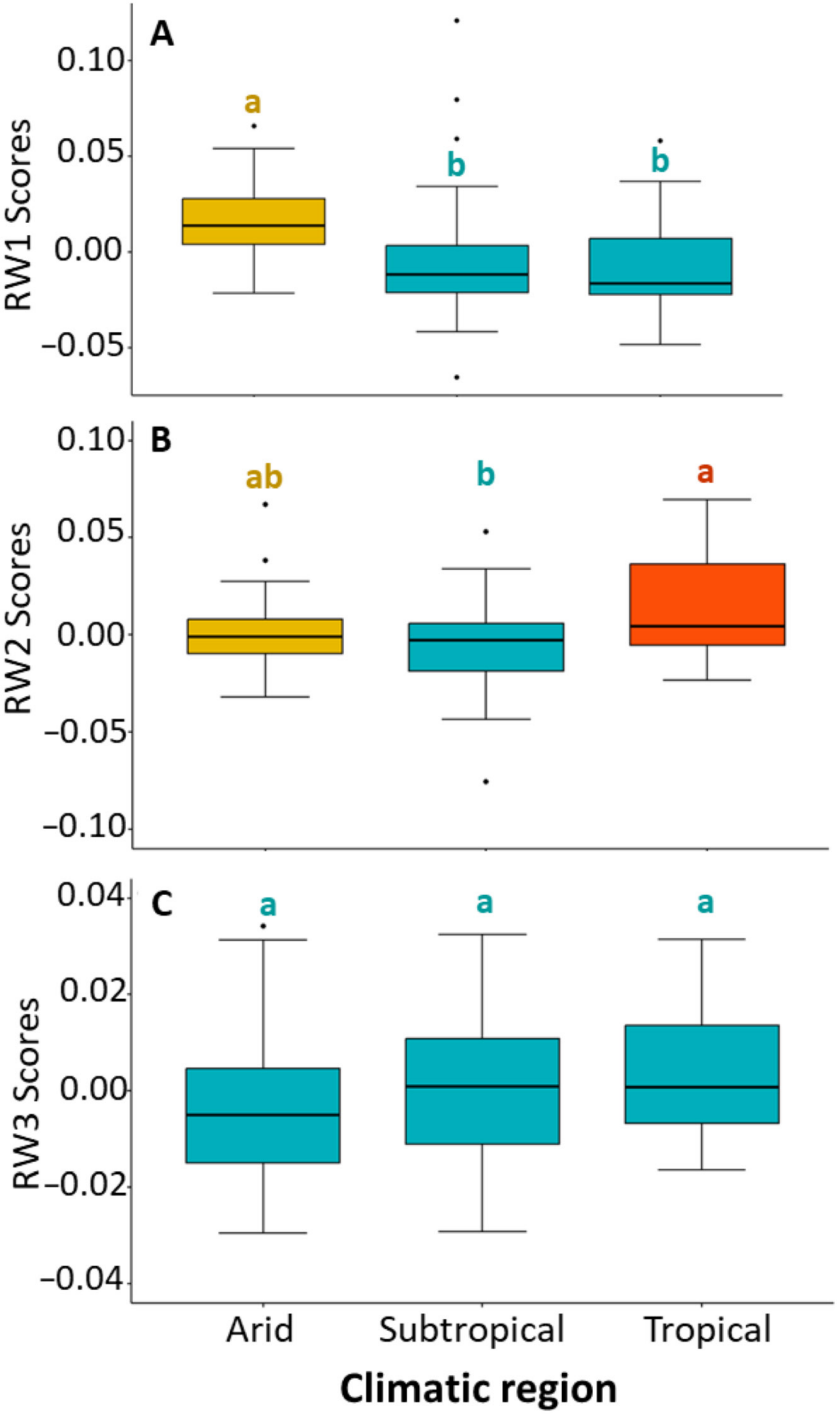
Boxplots showing shape variation of the female foretibia between climatic regions, as explained by relative warp (RW) scores: (A) RW1, (B) RW2, and (C) RW3. Letters above boxplots indicate statistically significant groups based on post hoc Tukey HSD tests. Boxplots show median values (black line) with first (lower hinge) and third (upper hinge) quartiles. Whiskers represent minimum and maximum values, while black dots signify outliers.

**FIGURE A1:**
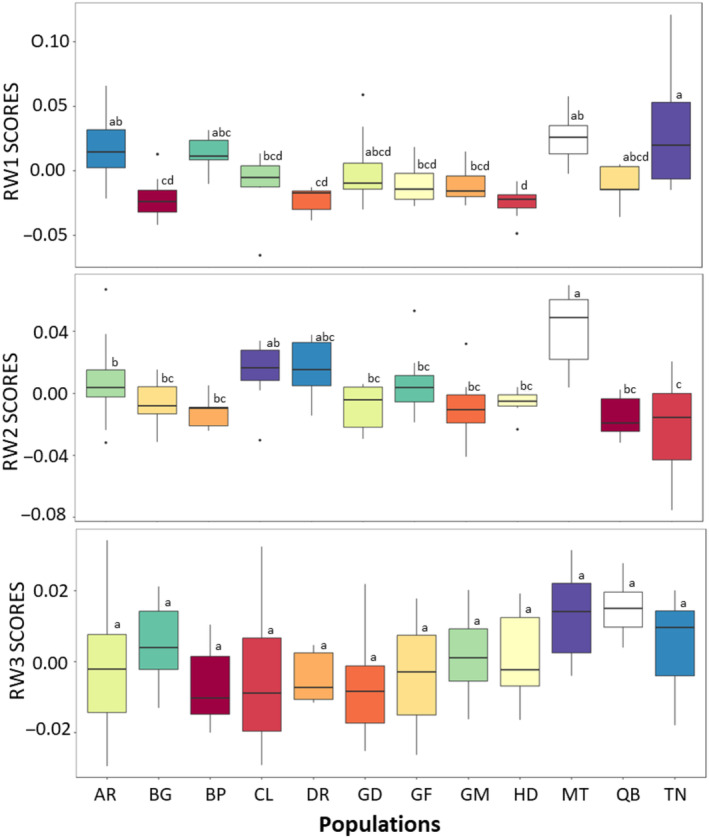
Boxplots showing differences between populations for RW1 (top), RW2 (middle) and RW3 (bottom) of the female foretibia. Insert letters denote statistically significant groups. Letters below plot are abbreviations for different populations (AR = Arumbera, BG = Biggenden, BP=Burt Plain, CL = Colosseum, DR = Durack, GD = Goondiwindi, GF = Grafton, GM = Graman, HD=Humpty Doo, MT = Mataranka, QB = Quambone, TN = Tenandra). Boxplots show median values (black line) with first (lower hinge) and third (upper hinge) quartiles. Whiskers represent minimum and maximum values, while black dots signify outliers.

For the relative warp analysis of female foretibia, the first 13 RWs each explain ≥ 1% of the variation in trait shape. The first three RWs collectively explained more than 65% of shape variation, with RW1 explaining 34%, RW2 explaining 24% and RW3 explaining 9%. For RW1, negative scores described foretibia that were thicker and with longer and sharper tibial teeth than positive scores (Figure 4). RW2 described the orientation of the teeth relative to the tibia, with negative scores describing backward pointing and positive scores describing forward pointing teeth (Figure 4). RW3 described subtle variation in the relative length of the 4th tibial tooth with negative scores associated with a more pronounced 4th tooth (Figure 4). Multivariate Analysis of Covariance (MANCOVA) controlling for CS found significant overall variation in the three RWs, both among populations (*F*_11,95_ = 4.46, *p* < 0.0001) and climatic regions (*F*_2,104_ = 4.84, *p* < 0.001). Univariate post hoc Tukey HSD tests detected differences between populations for RW1 and RW2 (Figure A1 in the Appendix 1) and between climatic regions for RW1 and RW2 (Figure 5).

**FIGURE 6:**
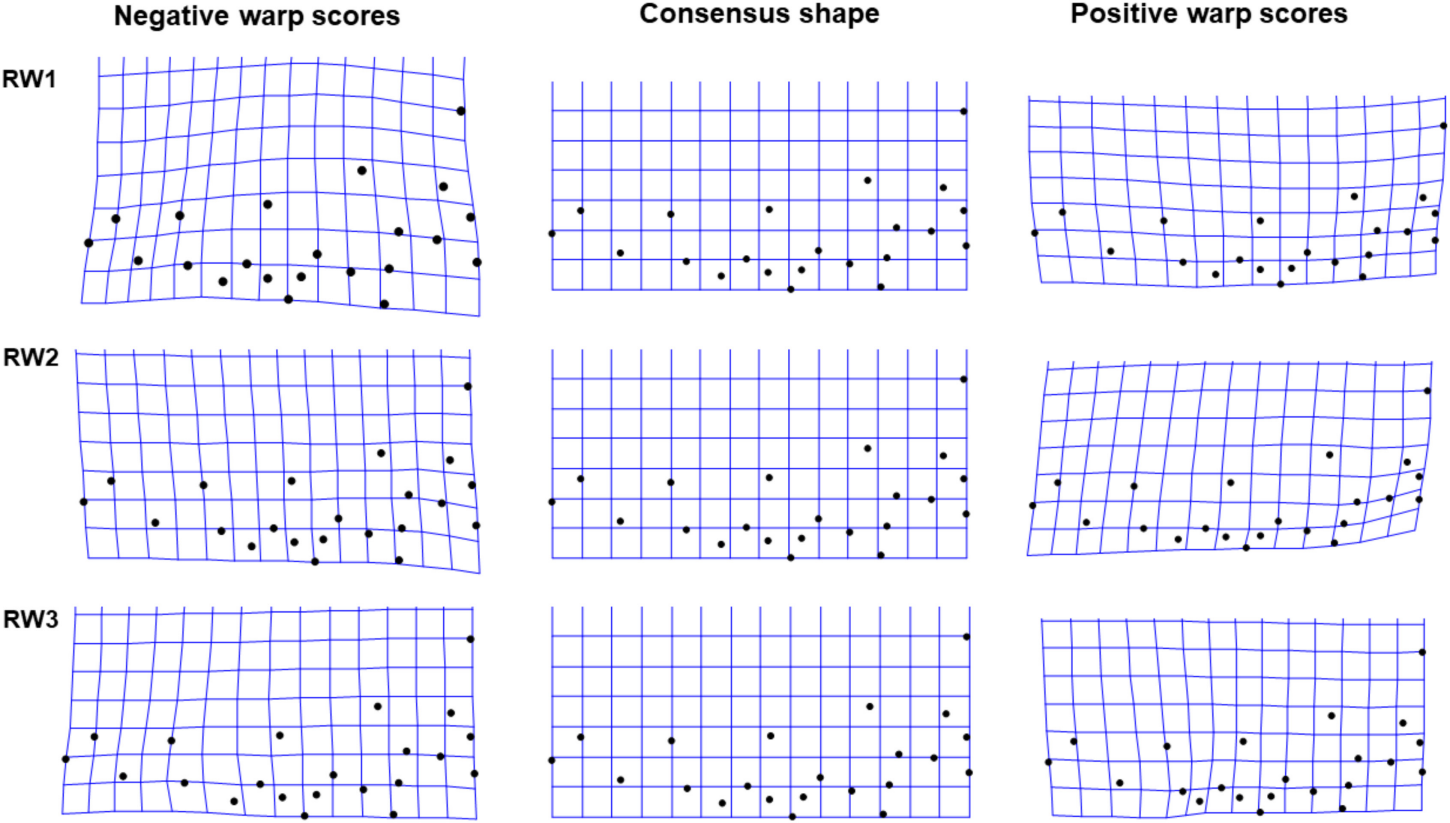
Thin plate splines showing shape variation as explained by RW1, RW2 and RW3 of the male foretibia. Black dots show position of landmarks. Top panel indicates RW1 shape variation, middle panel indicates RW2 shape variation, while bottom panel indicates RW3 shape variation.

**FIGURE 7:**
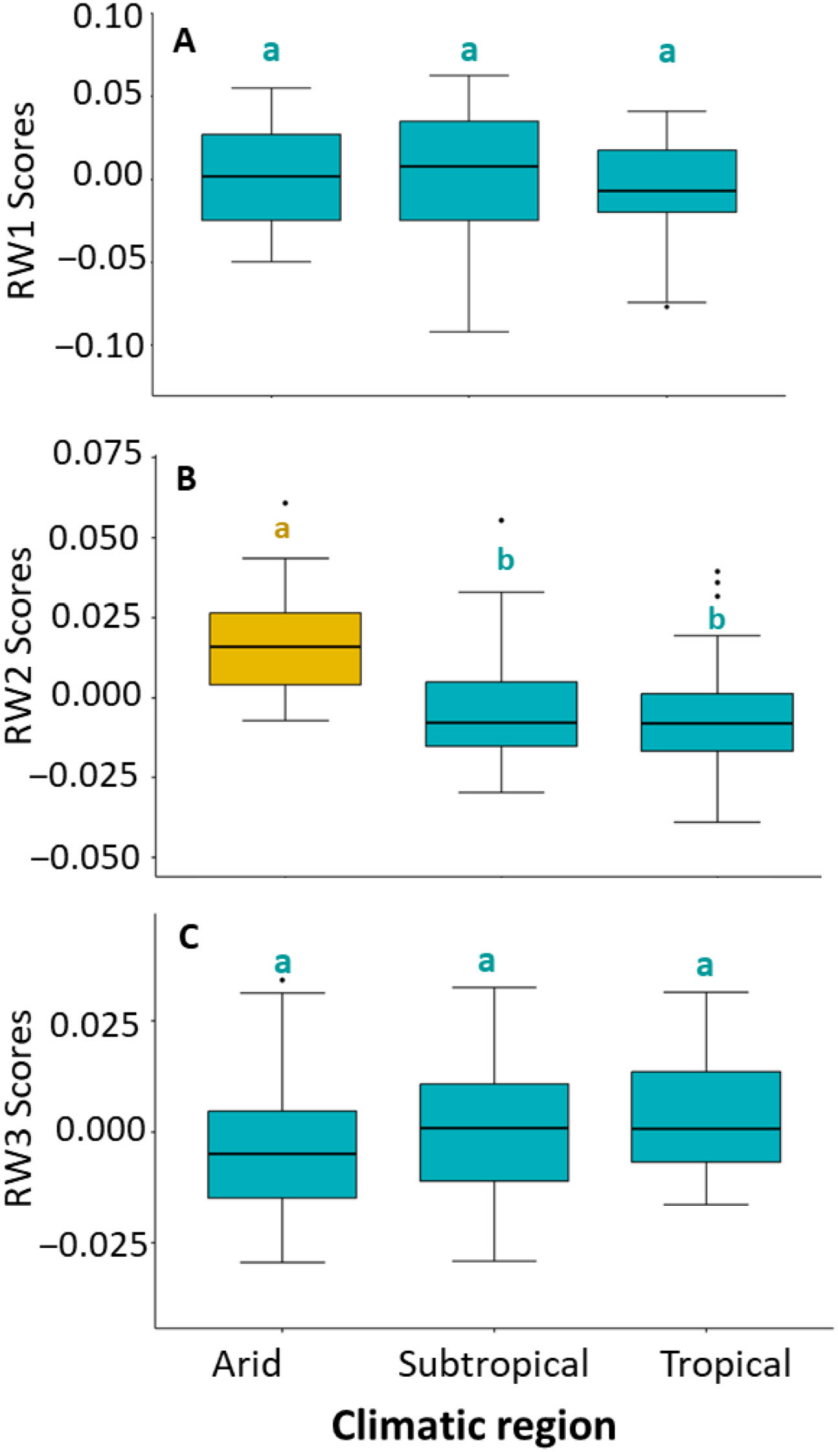
Boxplots showing shape variation of the male foretibia between climatic regions, as explained by relative warp (RW) scores: (A) RW1, (B) RW2, and (C) RW3. Letters above boxplots indicate statistically significant groups based on post hoc Tukey HSD tests. Boxplots show median values (black line) with first (lower hinge) and third (upper hinge) quartiles. Whiskers represent minimum and maximum values, while black dots signify outliers.

**FIGURE A2:**
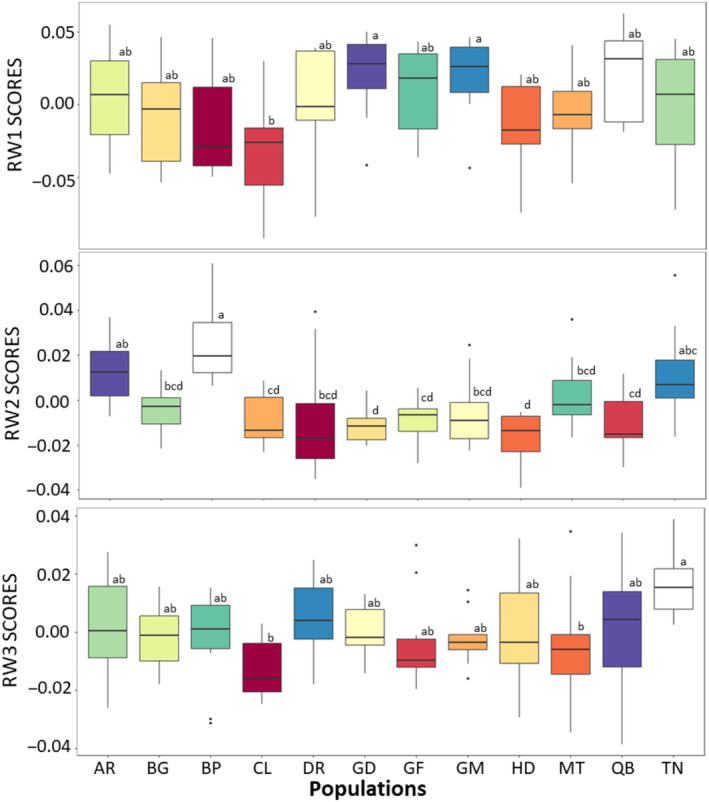
Boxplots showing differences between populations for RW1 (top), RW2 (middle) and RW3 (bottom) of the male foretibia. Insert letters denote statistically significant groups. Letters below plot are abbreviations for different populations (AR = Arumbera, BG = Biggenden, BP=Burt Plain, CL = Colosseum, DR = Durack, GD = Goondiwindi, GF = Grafton, GM = Graman, HD=Humpty Doo, MT = Mataranka, QB = Quambone, TN = Tenandra). Boxplots show median values (black line) with first (lower hinge) and third (upper hinge) quartiles. Whiskers represent minimum and maximum values, while black dots signify outliers.

Similarly, for the relative warp analysis of male foretibia, the first 12 RWs each explain ≥ 1% of the variation in trait shape. The first three RWs collectively explained more than 70% of shape variation in the male foretibial morphology, with RW1 explaining 48%, RW2 explaining 14% and RW3 explaining 11%. Positive scores on RW1 described slender tibiae with shorter tibial teeth, while negative scores described thicker tibia with longer tibial teeth (Figure 6). RW2 described variation in the angle between the first and second tibial tooth, while RW3 described variation in the shape of the 4th tibial tooth (Figure 6). Multivariate Analysis of Covariance (MANCOVA) controlling for CS found significant overall variation in the three RWs, both among populations (*F*_11,122_ = 3.13, *p* < 0.001) and climatic regions (*F*_2,131_ = 6.61, *p* < 0.0001). Univariate post hoc Tukey HSD tests indicated differences between populations in all three RWs (Figure A2 in the Appendix 1), and between climatic regions for RW2 only (Figure 7).

### 3.3 Population Structure and Genetic Diversity

**FIGURE 8:**
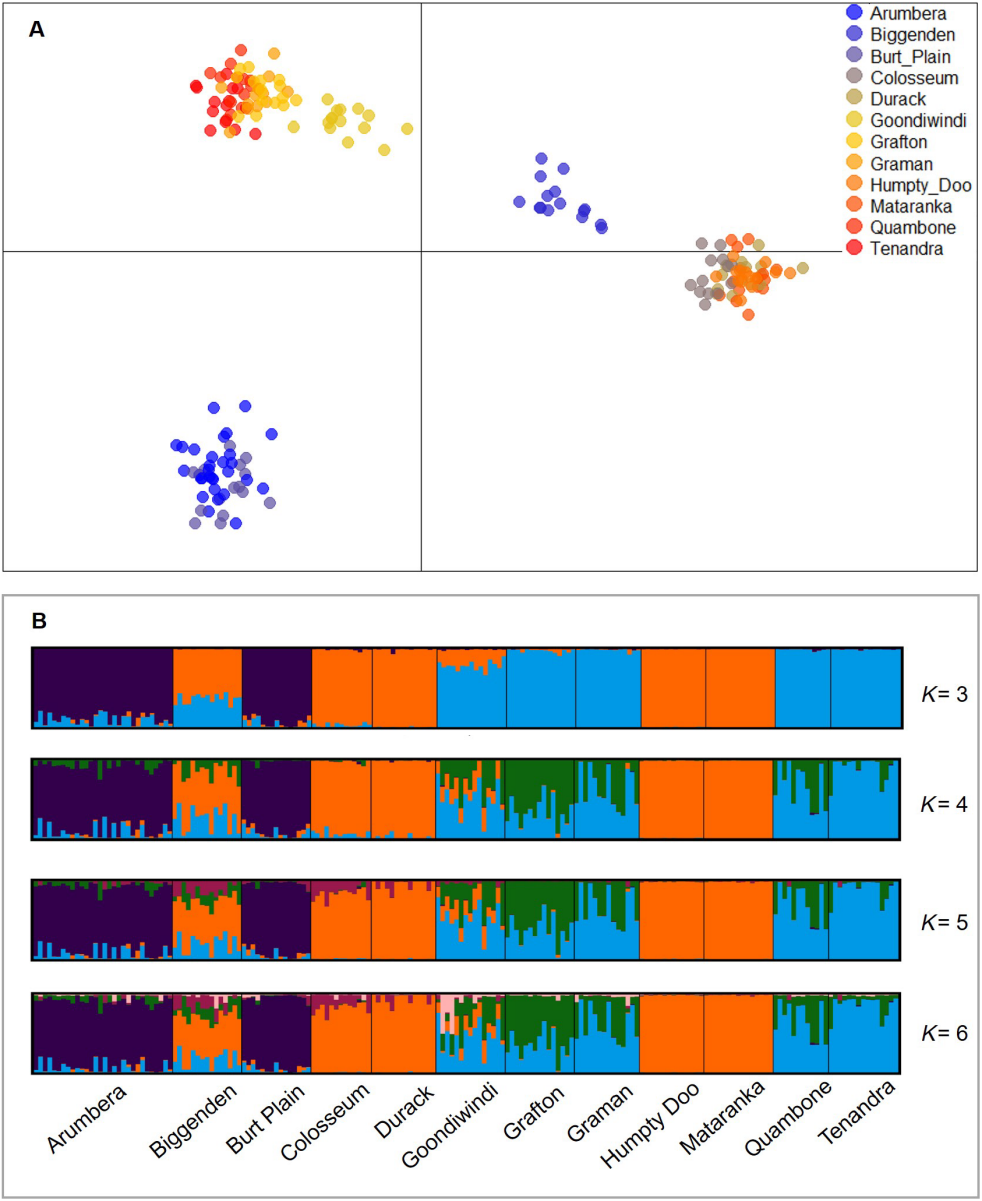
Clustering analysis results based on 1594 neutral SNP loci. (A) Scatterplot of discriminant analysis principal components (DAPC). (B) Bar chart of ancestry coefficients from the STRUCTURE analysis for varying values of *K*.

**FIGURE A3:**
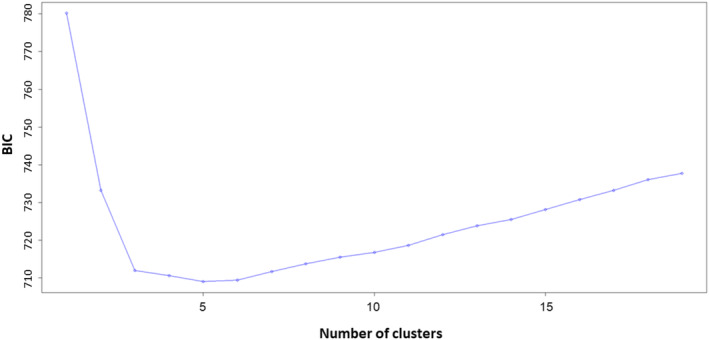
Bayesian Information Criterion (BIC) plot showing the optimal number of ancestral clusters (*K* = 5).

DArTseq Analytical Pipelines produced 28,734 SNP loci. After data filtering and screening for outlier loci, a dataset consisting of 1594 putatively neutral loci were retained for subsequent analyses (Table A2 in the Appendix 1). The DAPC analysis grouped populations into five distinct clusters (Figure 8). This was also the optimal number of genetic clusters based on the Bayesian Information Criterion (Figure A3 in the Appendix 1). Individuals from the two populations (Arumbera and Burt Plain) in central Australia were grouped into a single cluster. Individuals from central and northern NSW and south‐eastern QLD were split between four genetic clusters. Individuals from Goondiwindi and Biggenden each formed separate genetic clusters, while individuals from Colosseum were grouped into a cluster with all tropical region populations in northern WA and NT. Individuals from the remaining populations in this region formed their own separate single cluster. The analyses based on the Bayesian clustering program STRUCTURE produced similar groupings when the number of populations were set to between three and six, with the clearest groupings consisting of the two arid central Australian populations (Arumbera and Burt Plain), the tropical region populations in northern WA, northern NT and south eastern QLD (Biggenden, Colosseum, Durack, Humpty Doo and Mataranka) and the central and northern NSW and south‐eastern QLD populations (Goondiwindi, Grafton, Graman, Quambone and Tenandra) (Figure 8). However, the delta *K* statistic indicated that the optimal number of genetic clusters was two.

The level of genetic differentiation among populations was moderate to high (Global *F*_ST_ = 0.118) with pairwise *F*_ST_ values ranging from 0.000 to 0.220. All pairwise *F*_ST_ estimates were significantly different from zero, except between Arumbera and Burt Plain, and between Quambone and Graman (Table 2). There was a significant positive relationship between pairwise *F*_ST_ and geographical distance (Mantel *r* = 0.517, *p* = 0.003). Estimates of genetic diversity varied significantly between most populations (Table 1; Table A3 in the Appendix 1). Notably, the two populations (Biggenden and Colosseum) closest to the first release site (Townsville) had significantly higher genetic diversity than several populations that are more geographically separated from Townsville. However, there was no significant relationship between expected heterozygosity and distance to Townsville (*F*_1,10_ = 0.02, *p* = 0.896). Inbreeding coefficients were significantly positive across all populations, ranging from 0.073 to 0.144 (Table 1).

**TABLE 2:** Pairwise *F*_ST_ estimates between populations of *D. gazella*, based on 1594 neutral SNP loci.

Population	AR	BG	BP	CL	DR	GD	GF	GM	HD	MT	QB
BG	0.128*										
BP	0.000	0.132*									
CL	0.185*	0.024*	0.187*								
DR	0.202*	0.043*	0.206*	0.017*							
GD	0.089*	0.042*	0.097*	0.108*	0.126*						
GF	0.109*	0.085*	0.128*	0.169*	0.189*	0.026*					
GM	0.093*	0.079*	0.106*	0.162*	0.181*	0.010*	0.021*				
HD	0.209*	0.050*	0.213*	0.023*	0.010*	0.135*	0.188*	0.187*			
MT	0.206*	0.046*	0.209*	0.019*	0.005	0.133*	0.187*	0.185*	0.008*		
QB	0.094*	0.087*	0.109*	0.174*	0.195*	0.012*	0.020*	0.005	0.202*	0.198*	
TN	0.098*	0.101*	0.104*	0.187*	0.209*	0.025*	0.050*	0.018*	0.220*	0.216*	0.009*

* Significantly greater than zero at *p* = 0.05 after correction for multiple comparisons.

### 3.4 Phenotypic Divergence in Foretibia Shape

**FIGURE 9:**
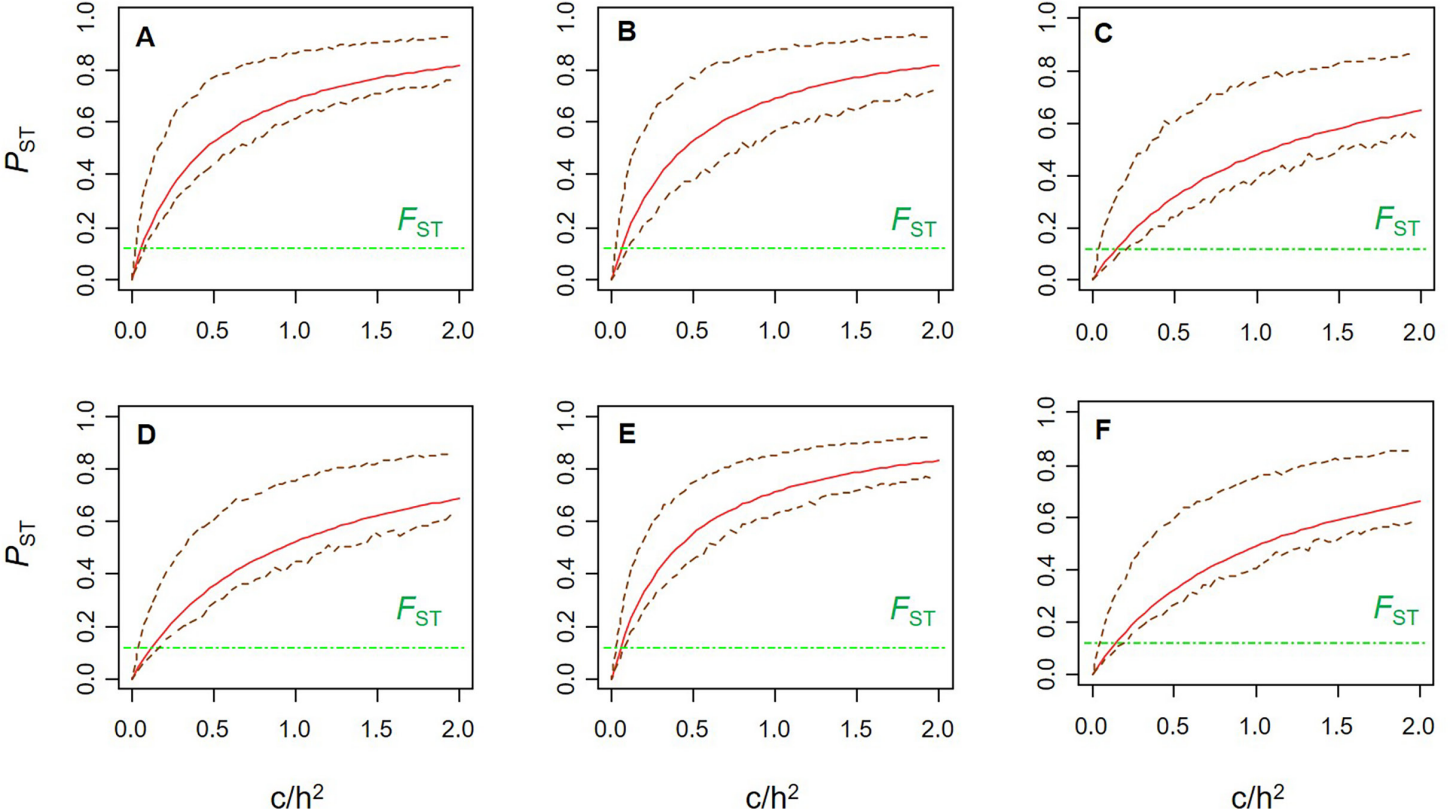
Comparison of global *P*_ST_ and *F*_ST_ for different relationships between the proportion of total variance assumed to be caused by additive genetic effects across populations (c) and heritability (*h* ^2^). When *P*_ST_ is much higher than *F*_ST_, and the lower 95% confidence interval of *P*_ST_ exceeds *F*_ST_ when *c*/*h* ^2^ < 1, it can be concluded that the *P*_ST_ is significantly higher than the *F*_ST_ (Brommer 2011). (A) Female RW1; (B) Female RW2; (C) Female RW3; (D) Male RW1; (E) Male RW2; (F) Male RW3. The horizontal dotted line represents the value of global *F*_ST_. The red line represents the global *P*_ST_, and the brown dashed lines represent the associated 95% confidence intervals.

Global *P*_ST_ values were higher than the global *F*_ST_ across a range of additive genetic variance scenarios for the first three RW scores in both females and males (Figure 9). Notably, for the RW scores in both sexes, the lower 95% confidence interval for global *P*_ST_ was higher than *F*_ST_ when the *c*/*h* ^2^ ratio was less than one. This provides evidence that *P*_ST_ is significantly higher than the *F*_ST_, and that this finding is robust (Brommer 2011). With the exception of RW1 in females, pairwise *P*_ST_ values were not significantly correlated with pairwise *F*_ST_ (Table 3).

**TABLE 3:** Relationships between pairwise *F*_ST_ and *P*_ST_ estimates.

	Female		Male	
Trait	Mantel *r*	*P*	Mantel *r*	*P*
RW1	**0.238**	**0.039**	−0.090	0.753
RW2	0.192	0.073	0.207	0.058
RW3	−0.013	0.544	−0.031	0.604

*Note:* Bold text indicates a significant relationship between pairwise PST and pairwise FST (*p* < 0.05).

## 4 Discussion

Evolutionary processes occurring after a species is translocated to a new location play a key role in determining the efficiency and effectiveness of a translocation programme (Briski et al. 2018; Li et al. 2019; Wright and Bennett 2018). This study examined morphological divergence and population genetic structure in a translocated dung beetle, in a bid to understand the evolutionary processes underlying a successful species' introduction. Using mitochondrial DNA sequence and SNP data, we show the presence of a single *Digitonthophagus* species within Australia. We provide evidence of high levels of genetic and morphological variation among populations, suggesting that both genetic drift and directional selection have played key roles during the establishment of this species across the Australian landscape. Furthermore, despite the rapid spread of *D. gazella* across Australia, it appears that gene flow is restricted across small to moderate spatial scales among established populations, highlighting the vulnerability of populations to local extinctions and loss of genetic diversity.

### 4.1 Species Characterisation and Distribution

The mitochondrial COI sequences suggest that only a single *Digitonthophagus* species (*D. gazella*) was introduced into Australia. All sequences obtained from the sampled populations formed a single monophyletic clade in a maximum likelihood phylogenetic tree along with two sequences of *D. gazella* sourced from Genbank. This view was further supported by the low sequence divergence among the sampled populations (1.3%), compared to high sequence divergences found between species within the genus *Digitonthophagus* and within the tribe Onthophagini. The low sequence divergence between sampled populations also falls within the range commonly found within species for COI (Hebert, Ratnasingham, and De Waard 2003). However, it should be noted that if another species, such as *D. namaquensis*, does occur at a low frequency in Australia, our sampling may have been insufficient to detect it.

The spread of *D. gazella* across Australia, beyond regions that were predicted based on climatic modelling, suggested that one of the several ‘strains’ introduced to Australia could have been a different species in the genus *Digitonthophagus*. The initial 1968 release of *D. gazella* was of a ‘tropical’ strain sourced from the tropical region east of sub‐Saharan Africa (Bornemissza 1976; Edwards 2007; Markin and Yoshioka 1998), while subsequent introductions of ‘cold’, ‘even rainfall’ and ‘gene pool’ were sourced from multiple regions across southern and eastern Africa (Bornemissza 1976; Edwards 2007; Génier and Davis 2017) to supplement genetic diversity of the founding populations. Based on our findings, it appears that these ‘strains’ represent different biotypes of *D. gazella* rather than different species, potentially each with a capacity to establish and colonise different climatic regions (Floate et al. 2017; Pokhrel, Cairns, and Andrew 2020). Unfortunately, we have been unable to source specimens of the different strains prior to their release, preventing us from mapping source strains to the different regions of Australia that *D. gazella* now inhabit.

The expansion of *D. gazella* beyond its range based on climatic modelling is most apparent in the arid regions of central Australia (Noriega et al. 2020; Pokhrel, Cairns, and Andrew 2020). The ability of *D. gazella* to persist in these regions may be due to both biotic and abiotic factors. First, *D. gazella* may be able to thrive in arid regions due to the provision of suitably moist conditions via irrigation systems (Floate et al. 2017). However, Noriega et al. (2020) note that some of the arid regions are not entirely irrigated, suggesting that factors other than irrigation could be contributing to the survival of *D. gazella*. Second, *D. gazella* could be occupying a more diverse range of habitats due to the absence of predatory fauna (Noriega et al. 2020). In Australia, there are no notable natural enemies of *D. gazella*, except for the invasive cane toad (*Rhinella marina*) (González‐Bernal et al. 2013). The absence of cane toads in arid regions (Selechnik et al. 2017) could, therefore, partly explain the persistence of *D. gazella* in these regions. Lastly, the survival of *D. gazella* in warmer regions may have been facilitated through phenotypic plasticity; ancestral plasticity and the evolution of plastic responses appear to have contributed to local adaptation and population differentiation during the invasion and range expansion of introduced *O. taurus* populations in the United States (Rohner and Moczek 2020). Thus, Rohner and Moczek (2020) found evidence of clinal variation across a variety of traits: northern populations were found to exhibit shorter development time, smaller body size, disproportionately larger wings and broader foretibia than southern populations. Similarly, *O. taurus* translocated for biocontrol purposes was found to have the capacity to adjust its cuticular hydrocarbon (CHC) profile when exposed to desiccation stress along a south–north climatic gradient of increasing temperatures in Western Australia (Leeson et al. 2020).

The ability for introduced species to expand their ranges into novel environments is generally recognised as being dependent on phenotypic plasticity (Ghalambor et al. 2007; Jardeleza et al. 2022; Richards et al. 2006). Evidence of plasticity's role in range expansion comes from a variety of taxa. For example, the cane toad (*R. marina*) was introduced to Australia for the control of sugar cane beetle (*Dermolepida albohhirtum*) but subsequently became invasive (Phillips et al. 2006). Cane toads exhibit both phenotypic plasticity and genetic adaptation in leg length, a trait associated with increased dispersal ability which has facilitated their spread across the continent (Phillips, Brown, and Shine 2010; Phillips et al. 2006). Likewise, the European whitefish (*Coregonus lavaretus*), translocated to six new lakes in Scotland as part of a conservation effort to protect the species from potential extinction (Adams et al. 2014), displayed changes in body shape in response to the different ecological conditions inherent to these novel lakes (Crotti et al. 2021). It is possible therefore, that phenotypic plasticity could have facilitated the spread of *D. gazella* into the arid regions of Australia. In support of this hypothesis, *D. gazella* has been recorded from the extremely arid regions of the Tatacoa Desert in Colombia, and its survival there has been attributed to the species' plastic responses to harsh conditions (Noriega 2016; Noriega et al. 2020).

### 4.2 Population Genetic Structure

A key finding from this study was the high genetic differentiation among populations, suggesting that *D. gazella* in Australia has restricted gene flow and that populations are genetically isolated over moderate spatial scales (74–500 km). A positive relationship between pairwise *F*_ST_ and geographic distance indicates that genetic differentiation increases as geographic distance increases. This was further indicated by the geographic patterns shown by the DAPC analysis, which grouped populations into five genetically distinct clusters. These clusters represent individuals from each of the main climatic regions, although some populations were grouped with another region (e.g., Colosseum) or formed their own cluster (e.g., Goondiwindi and Biggenden).

It is clear that *D. gazella* underwent a rapid range expansion since it was first introduced to Australia, which has been likely aided by its high dispersal rate (Seymour 1980), high reproductive rate (Blume and Aga 1975) and short generation time (Pokhrel, Cairns, and Andrew 2020; Whipple et al. 2012). The rate of spread was so rapid that in less than two years, *D. gazella* had become established in the northern tropical regions from Townsville, Queensland to Broome, Western Australia (Edwards 2007). Ultimately, *D. gazella* expanded its range to arid regions in inland Australia (Floate et al. 2017). These range expansions across large geographic distances provided the opportunity for significant geographic isolation between populations and subsequent genetic differentiation. Serial founder effects and bottlenecks that occurred during the range expansion may have further compounded genetic differentiation. When a founder event arises during a wide range expansion, genetic drift occurs rapidly as the resulting populations tend to have relatively lower genetic diversity than their ancestral founder population (Slatkin and Excoffier 2012). Consistent with this pattern, we found some evidence of higher genetic diversity in populations close to the initial release site (Townsville) than those further away. However, the relationship between genetic diversity and geographic distance from the first release site was non‐significant. This may reflect a history of multiple, subsequent releases at different sites (southern Australia and eastern New South Wales) between 1970 and 1984 (Edwards 2007), of which the precise locations are not known. This complex colonisation history may have also contributed to the patterns of population structure we observed. Even so, we would expect such historical patterns to break down quickly under high gene flow over many generations, further suggesting that gene flow among established populations is restricted.

### 4.3 Morphological Divergence and Adaptive Evolution

In addition to high genetic divergences between populations in neutral genetic markers, we found evidence of significant variation among populations and among climatic regions, in an ecologically important trait, foretibia morphology, in both sexes. In paracoprids, the foretibia is a shovel‐shaped stout tool utilised mainly for digging tunnels in the soil and burying dung underground (Linz, Hu, and Moczek 2019; Scholtz, Davis, and Kryger 2009). The foretibia has enabled dung beetles to inhabit and ecologically adapt to a niche that cannot be accessed by other insect taxa (Linz, Hu, and Moczek 2019). Both the male and female are active in dung provisioning for brooding (Simmons and Fitzpatrick 2019). Females spend more time digging tunnels and packing brood balls (Bornemissza 1970) and generally have thicker, more robust foretibia than males (Simmons and Fitzpatrick 2019). Males also use their foretibia when assisting females with packing the brood balls (Bornemissza 1970; Simmons and Ridsdill‐Smith 2011).

Factors such as temperature‐dependent nesting behaviour, moisture and/or type of soil are known to drive both intraspecific and interspecific divergence in foretibia morphology of tunnelling dung beetles (Macagno, Moczek, and Pizzo 2016; Macagno et al. 2018; Rohner and Moczek 2020; Simmons and Fitzpatrick 2019). In our study, we found that for females, populations from the inland desert region had thinner, elongated foretibiae, while populations from the tropical and subtropical climatic regions exhibit thicker and shorter foretibia (RW1). Our findings are consistent with those of Rohner and Moczek (2020), who found a plastic latitudinal variation in foretibia morphology in exotic populations of *O. taurus* in North America. Populations from the relatively cooler northern region had a robust foretibiae with a shorter base, while populations from the relatively warmer southern region had a narrower and more arched foretibiae with a longer base (Rohner and Moczek 2020). When studying maternal behaviour in *O. taurus*, Macagno et al. (2018) found that introduced populations exhibited maternal behavioural plasticity in brood burial depth for thermoregulatory purposes. Therefore, variation in foretibia morphology may be associated with adaptation to temperature‐dependent maternal care which affects how deep the brood burial nest needs to be dug in different climatic regions (Rohner and Moczek 2020).

Comparing *P*_ST_ with *F*_ST_ suggests that directional selection may be contributing to the foretibia morphological divergences seen among Australian *D. gazella* populations. With the exception of RW1 in females, pairwise *F*_ST_ and *P*_ST_ were not correlated. Moreover, global *P*_ST_ estimates for all RWs were higher than global *F*_ST_ over a wide range of *c*/*h* ^2^ ratios, indicating that the degree of differentiation in morphology was significantly higher than the degree of differentiation occurring at neutral SNP markers distributed across the genome. Together, these analyses suggest directional selection may be contributing to the morphological divergences among populations and that these populations may be locally adapted to some extent. An alternate possibility is that the morphological variation we observed existed prior to the introduction to Australia and that the differences among Australian populations reflect the colonisation history of different strains. Indeed, divergence in horn allometry between geographically isolated populations of *O. taurus* in Western Australia and North Carolina (USA) has been suggested to result from pre‐existing variation in horn allometry in the native range of the species (Moczek et al. 2002), and the same could be true for divergence in foretibia that we have observed in *D. gazella*. However, the lack of a relationship between genetic and phenotypic divergence in the Australian populations argues against this possibility.

### 4.4 Implications for Biological Control Management

The evolutionary mechanisms that have allowed *D. gazella* to expand its niche across its Australian range have important management implications. This study demonstrates that a strong population structure has developed rapidly, within a period of 50 years. After such a wide range expansion, introduced populations become geographically isolated and consequently susceptible to genetic drift and the loss of genetic diversity. Our finding of reduced genetic diversity in some populations and directional selection acting on an ecologically relevant trait highlights the importance of maximising genetic variation in stock populations prior to translocation, to offset the loss of genetic variation and allow local adaptation when populations colonise new areas (e.g., Binks, Kennington, and Johnson 2007).

Comparing levels of genetic diversity in Australian *D. gazella* populations to levels in the native range was beyond the scope of this study. However, we expect that the genetic diversity in the Australian populations will be significantly lower than in the source populations and recommend that future studies investigate whether this is the case. Evidence of low genetic diversity in some Australian populations argues for the need for re‐introductions/supplementation of *D. gazella* across its Australian range. However, care should be taken when conducting these interventions to avoid introducing maladapted alleles to potentially locally adapted populations (Huff et al. 2011). This could be best achieved by restricting translocations to among populations within the same geographic region and areas with similar environments. Additional studies on other introduced dung beetle species are also warranted in order to elucidate whether the patterns observed within *D. gazella* extend to other translocated dung beetles.

## Author Contributions

**Boikhutso Lerato Rapalai:** conceptualization (equal), data curation (lead), formal analysis (lead), investigation (lead), methodology (lead), writing – original draft (lead), writing – review and editing (equal). **Leigh W. Simmons:** conceptualization (equal), funding acquisition (supporting), investigation (supporting), methodology (equal), project administration (equal), resources (equal), supervision (equal), writing – original draft (supporting), writing – review and editing (equal). **Theodore A. Evans:** conceptualization (equal), funding acquisition (lead), project administration (equal), supervision (equal), writing – review and editing (equal). **W. Jason Kennington:** conceptualization (equal), data curation (supporting), formal analysis (supporting), funding acquisition (supporting), investigation (supporting), methodology (supporting), project administration (equal), resources (supporting), supervision (equal), writing – original draft (supporting), writing – review and editing (equal).

## Conflicts of Interest

The authors declare no conflicts of interest.

## Data Availability

Data and scripts required to reproduce the analysis described herein are available from The University of Western Australia Research Repository: http://doi.org/10.26182/v181‐ry58.

## Appendix 1

### 1.1

Here we provide additional Tables and Figures to support the main analyses.

**TABLE A1:** Percentage COI sequence divergence between *D. gazella* individuals sampled in this study (sampled populations) and between GenBank sequences at different taxonomic levels.

	Mean	Minimum	Maximum	Pairwise comparisons
Sampled populations	1.3	0	2.8	1770
*D. gazella* Genbank	4.7	2.1	7.5	6
Genus *Digitonthophagus*	11.6	11.2	11.8	10
Tribe Onthophagini	14.0	10.8	17.3	28

**TABLE A2:** Filtering steps used for producing the SNP data for analysis.

Filtering step	Loci	Individuals	% loci retained
Original data	28,734	188	—
Locus call rate (0.95 threshold)	5170	188	18.0
Individual call rate (0.85 threshold)	5170	187	18.0
Monomorphs	5069	187	17.6
Min allele frequency (0.05 threshold)	1816	187	6.3
One SNP per sequence	1630	187	5.7
Non‐neutral loci	1594	187	5.5

**TABLE A3:** Results of Dunn's non‐parametric all pair comparison test used to detect significant differences between populations, based on expected heterozygosity (*H*_e_) calculated using 1594 neutral SNP loci. Multiple comparisons were corrected with the Benjamin‐Hochberg method.

	AR	BG	BP	CL	DR	GD	GF	GM	HD	MT	QB
BG	**0.000**										
BP	0.335	**0.000**									
CL	0.133	**0.009**	**0.010**								
DR	0.353	**0.001**	**0.049**	0.594							
GD	0.054	**0.027**	**0.003**	0.693	0.349						
GF	**0.027**	**0.000**	0.250	**0.000**	**0.001**	**0.000**					
GM	0.389	**0.000**	0.894	**0.016**	0.068	**0.004**	0.195				
HD	0.556	**0.000**	0.107	0.370	0.729	0.198	**0.004**	0.146			
MT	0.953	**0.000**	0.300	0.149	0.369	0.064	**0.023**	0.365	0.592		
QB	**0.023**	**0.000**	0.224	**0.000**	**0.001**	**0.000**	0.942	0.173	**0.004**	**0.020**	
TN	**0.007**	**0.000**	0.102	**0.000**	**0.000**	**0.000**	0.644	0.070	**0.001**	**0.005**	0.689

*Note:* Bold text denotes pairwise values that were significantly different (*p* < 0.05). Letters indicate abbreviations for different populations.

Abbreviations: AR = Arumbera, BG = Biggenden, BP=Burt Plain, CL = Colosseum, DR = Durack, GD = Goondiwindi, GF = Grafton, GM = Graman, HD=Humpty Doo, MT = Mataranka, QB = Quambone, TN = Tenandra.
